# Association Between Dental Caries Prevalence and Stress Levels in Japanese Children

**DOI:** 10.7759/cureus.31074

**Published:** 2022-11-04

**Authors:** Reiko Nakano, Tomoko Ohshima, Yoko Mukai, Akihisa Tsurumoto, Nobuko Maeda

**Affiliations:** 1 School of Dental Medicine, Department of Oral Microbiology, Tsurumi University, Yokohama, JPN; 2 School of Dental Medicine, Department of Community Dentistry, Tsurumi University, Yokohama, JPN

**Keywords:** dmf, cariogenic bacteria, salivary stress proteins, dental caries, mental stress

## Abstract

Introduction

Early life stress (ELS) caused by abuse and bullying has increased dramatically, however, effective means for accurate detection have not been found. Some decades ago, an association between stress and dental caries was suggested. However, even now, stress is not recognized widely as a potential risk factor for caries. Therefore, the purpose of this study was to verify the possible effects of stress by comparing them to the effects of saliva factors and the microorganisms that pose a general caries risk.

Methods

We conducted cross-sectional observation research on 30 children with mental problems, diagnosed as ‘stressed,’ and 30 age-matched unstressed children in the same elementary school. An oral examination (dental caries diagnosis) and an oral environment survey (saliva test) were carried out in 2007. Further, the concentration and activity of salivary stress proteins were measured. All variables were statistically analyzed using the Mann-Whitney U test, correlation, and multivariate analysis.

Results

The dental caries experience ratio was significantly higher in the stress group, and only the concentration of CgA, a salivary stress protein, showed a significant difference. Unexpectedly, we did not detect any differences in the rates and counts of cariogenic bacteria or salivary buffering activity. Binomial logistic regression analysis only showed significance in the presence or absence of ELS.

Conclusion

Stress factors may have a stronger influence on caries development in the stress group than in the general caries risk. Therefore, long-term stress, causing changes in the children's bodies, might hint at important factors leading to the development of dental caries.

## Introduction

Dental caries is known as the most universal infectious disease that has plagued mankind in history. *The Streptococcus mutans *group is one of the most prevalent species of the genus *Streptococcus* in the oral commensal flora but compared with other oral *Streptococci,* the frequency of detection is generally low and individual differences are large [1]. The cariogenic factors of *Mutans streptococci* are adhesion to tooth enamel, insoluble polysaccharide production, excessive acid production, and acid resistance [2].

WHO set the target to reduce the number of caries experience teeth in 12-year-olds, indicated as DMFT (decayed, missing, filled teeth), emphasizing D (decayed teeth) [3]. In this connection, based on the Health Promotion Law, Japan introduced ‘Health Japan 21’ with the goal of <1.0 caries experience teeth on average per 12-year-old by 2022. Especially in Japan, the number of decayed teeth per 12-year-old was 0.3 in 2005 and 0.2 in 2016. Being diagnosed with a caries risk might promote oral hygiene management and contribute to caries prevention efforts. The current caries risk diagnosis is based on an investigation of microbial factors (*Mutans streptococci*, *lactobacilli,* and yeast) [4] using saliva and on host factors for caries resistance utilizing the secretion level and buffering capacity of saliva and carbohydrate eating habits [5].

Recently, there are reports that social disparity in developed countries is related to health disparity in both adults and children. It has been known for a long time that social disparity leads to chronic mental stress and that immunity is reduced by stress, contributing to health deterioration [6]. Another study suggested that childhood abuse could have longitudinal adverse effects on dental health at an older age [7]. The mechanism of this phenomenon has been clarified at the tissue, humoral factor, and cellular levels. Mental stress is transmitted to the hypothalamus via the cerebral cortex and the limbic system, and glucocorticoids (cortisol) are released by the activated Hypothalamic-Pituitary-Adrenal system (HPA-system) [8]. This response, called brain-immune interaction, suppresses the activity of immune cells, decreases cytokine production, and accelerates the atrophy of central and peripheral lymphoid tissues [9]. Thus, as stress affects the health condition of the whole body, it is also a possible factor for damaging oral cavity health. Only from 1980 until around 2010, the association between stress and caries has been verified both in human and animal studies [10]. There are also papers suggesting an association between dental caries and child abuse including neglect [11]. However, even now, stress does not receive wide recognition as a caries risk. The reason is that most studies were conducted before stress and child abuse were recognized as big social problems and the number of related papers was not large enough to be sufficiently verified. A relationship between stress and caries was also reported in 2010 [12], however, both supporting and opposing theories continue. A 2018 review stated that research still does not provide sufficient evidence for a final conclusion [13]. Therefore further studies are necessary.

In recent years, domestic violence and child abuse have become global social problems [14]. Not only aggressive physical abuse but also mental abuse, such as neglect in the home environment, seem to result in a lot of chronic early life stress (ELS) [15].

In addition, suicides due to bullying at school show no sign of decline, and children are living with various mental problems. School teachers, especially nursing teachers from the school infirmary, are able to notice and respond to such changes in children's minds. It is important for school nurses to deal with students in collaboration with other teachers and parents [16]. School infirmaries are always set up in Japanese elementary schools and junior high schools and play a central role in school health activities. Especially in the health management of children, they function as counselors who can discuss a wide range of problems spanning from worries about school life or career paths to mental problems and troubles at home [17]. By sharing information about a child's daily life with other teachers in the school, the nursing teachers can understand the child’s mental and physical condition and quickly notice changes in the behavior or state of stress [18]. However, the excessive burden on nursing teachers fulfilling various tasks is a constant concern.

Currently, the caries incidence rate in children is declining year by year in developed countries including Japan [19]. On the other hand, there are many examples of a high caries incidence rate in children living in complicated family environments [20]. Therefore, caries could be a marker for the detection of abuse-induced stress. However, awareness among dentists is very low. One possible reason is, that it is not clearly understood how caries develops after stress.

In this study, we investigated the presence or absence of stress (group), subjective stress (stressor, stress response, self-efficacy), saliva resistance to caries (saliva buffering capacity, saliva secretion level), cariogenic bacteria (*Mutans streptococci* class, *Lactobacilli* class, *Candida* class, presence of caries-related microorganisms), and objective stress (CgA_class, amylase_class, cortisol_class) in order to verify the relationship between stress and caries in children.

## Materials and methods

Subjects

This study was approved by the Ethics Review Committee of Tsurumi University School of Dental Medicine (Approval number 947) with the written informed consent of the children's parents and all staff of the school under examination.

A total of 166 children from first to fifth grade of an elementary school in A-city in Gunma prefecture, Japan, with the classification shown in Table 1, were selected. Thereof, 30 children (including those from child protection facilities and from mother and child dormitories) who appealed repeatedly to a nursing room with problems or got long-term counseling from a nursing teacher were classified as a group with problems and in a stressful environment (stress group). The causes for stress in the stress group were considered trouble in the home environment or friendship trouble, including bullying or mental stress. A control group, matching the stress group in number, age, and sex, was set up by randomly selecting children who were not part of the stress group. Details of both groups are shown in Table 2.

**Table 1:** Number of children of an elementary school in A-city, Gunma prefecture, Japan

School grade	Total number	Male	Female
1st grade	34	18	16
2nd grade	26	13	13
3rd grade	36	19	17
4th grade	37	19	18
5th grade	33	16	17

**Table 2:** Number of children in the stress group and control group

School grade	Total number	
	Stress group (male: female)	Control group (male: female)
1st grade	8 (5: 3)	8 (5: 3)
2nd grade	4 (2: 2)	4 (2: 2)
3rd grade	3 (2: 1)	3 (2: 1)
4th grade	13 (6: 7)	13 (6: 7)
5th grade	2 (2: 0)	2 (2: 0)

Examination items and method

Oral Cavity Examination

We conducted an examination of the oral disease condition of each child, and the caries experience tooth ratio (decayed and filled permanent teeth as DF and of primary teeth as df) was calculated.

Oral Environment Examination

Microbial test: Saliva collected prior to lunch was used as a sample. *Mutans streptococci* were cultured at 37℃ for 48 hours using Dentocult SM Kit (Oral Care Inc., Tokyo, Japan), and genus *Lactobacilli* were cultured at 37℃ for four days using Dentocult LB Kit (Oral Care Inc.). The bacterial quantity (CFU/mL) was determined by comparing the colony density with the model chart (http://dentocult.jp/120530.pdf) and classified according to the manufacturer’s instructions (Table 3). For genus *Candida*, the tongue dorsum was swabbed 10 times with a sterile cotton swab and streaked onto the surface of the ChromoAgar® Candida medium (Kanto Chemical Co., Inc., Tokyo, Japan) and cultured at 30 °C for 48 hours. The colonies were counted (CFU/mL) and classified.

**Table 3:** Classification of measured values

Class	0	1	2	3
Mutans streptococci (log CFU/ml)	<4.0	4.0-5.0	5.0-6.0	>6.0
Genus Lactobacilli (log CFU/ml)	<3.0	3.0-4.0	4.0-5.0	5.0-6.0
Genus Candida (log CFU/ml)	0	0-1	1-2	2-3
pH after the reaction	4.0-4.8	5.0-5.5	5.8-6.5	
Saliva secretion level (ml/min)	< 0.5	0.5 - 0.8	0.9 - 1.1	>1.2
CgA (pmol/ml)	<2.0	2.0 - 4.0	4.0 - 6.0	>6.0
Amylase (kIU/ml)	<40	40 – 80	80 - 120	>120
Cortisol (ng/ml)	<0.09	0.09 - 0.11	0.11 - 0.13	>0.13

Measurement of saliva buffering capacity: Using CAT 21 Buf® (J. Morita Corp., Tokyo, Japan), saliva, collected in a test tube, was judged on the color chart, and classified according to the manufacturer’s recommendation (Table 3).

Measurement of saliva secretion level: Saliva, stimulated by chewing paraffin gum for three minutes, was collected using a measuring cylinder and then classified (Table 3).

In order to evaluate stress subjectively, a questionnaire survey was conducted using the DSS-K Health Survey Sheet (Stress Management for Kids) (Chao 2017) with content easy to understand. DSS-K is a scale group consisting of the daily hassles scale, the stress response scale, and the stress management self-efficacy scale. Responses to 12 stressor items (cause of stress), 13 stress reaction items (result of stress), and 10 self-efficacy items (self-confidence to resolve or confront stress), were calculated as points and totaled. A high score in stressor and stress response with a low score in self-efficacy indicates high stress levels.

Concentration and Activity Measurement of Stress Substances in Saliva

Stimulated saliva was used as a sample. Chromogranin A (CgA) was measured with a microplate absorbance reader at 490 nm using the commercially available human CgA EIA Kit YK 070 (Yanaihara Institute Inc, Fujinomiya, Japan). For amylase, an enzyme activity of 20 μl saliva was measured using a commercially available salivary amylase monitor (Nipro, Osaka, Japan). Salivary cortisol was measured on a microplate absorbance reader at 650 nm using the commercially available Cortisol EIA Kit (Oxford Biomedical Research Inc., Rochester Hills, MI).

Statistical analysis was performed and described by classifying the concentrations and activities into continuous variables or classes (Table 3). Regarding salivary proteins, quartiles were calculated and values <Q1 were set as class 0, values of Q1<Q2 as class 1, values Q2<Q3 as class 2, and values Q3 or larger were set as class 3.

Statistical analysis

Using SPSS 14.0 J (SPSS Japan Inc., Tokyo, Japan), analysis was performed using the Mann-Whitney U test for comparison between the two groups, Spearman's rank correlation coefficient for correlational analysis, and binomial logistic regression analysis for the relationship analysis of DF (df) tooth ratio, and multiple items (groups, stressor, stress response, self-efficacy, saliva buffering capacity, saliva secretion level, *Mutans streptococci* class, *Lactobacilli* class, *Candida* class, CgA_class, amylase_class, cortisol_class). Only the two affiliated groups (stress or control) were category variables; all other 11 items were continuous variables.

## Results

Oral examination

From the elementary school examined, children from first to fifth grades, 30 each in the stress group and the control group were selected, and the caries experience tooth ratio was compared. The df, DF, as well as total df and DF tooth ratio (Table 4), were all significantly higher in the stress group than in the control group.

**Table 4:** Comparison of caries experience ratio between the stress and control groups Mean ± SD

	Control group	Stress group	p-value
df tooth ratio (%)	11.74±21.6	31.64±25.52	<0.001
DF tooth ratio (%)	0.47±1.47	6.74±12.02	0.028
df+DF tooth ratio (%)	8.29±13.61	22.30±19.75	<0.001

Oral environment

The microbial test results of the control group and the stress group showed no difference in the class frequency with regard to* Mutans streptococci* and genus *Candida* (Table 5). Regarding genus *Lactobacilli*, class 1 and class 2 were found only in the stress group, but there was no statistically significant difference between the class mean value and the class frequency in both groups (Table 5).

**Table 5:** Class average of microbial factors, saliva, and stress awareness (Mean ± SD) a) ‘not at all’ answers show the minimum value of 12 points b) ‘not at all’ answers show the minimum value of 10 points The max. score of stressor, stress response, and self-efficacy were 60, 48, and 40.

		Control group	Stress group	p-value
Microbial factors	Mutans streptococci	0.07±0.25	0.07±0.25	-
	Genus Lactobacilli	0.00±0.00	0.23±0.57	0.157
	Genus Candida	0.27±0.74	0.43±0.82	
Saliva	Saliva buffering capacity	5.74±0.59	5.92±0.60	
	Saliva secretion level	4.37±0.67	4.76±1.69	
Stress awareness	Stressor ^a)^	22.03±9.15	23.97±7.36	0.105
	Stress response ^a)^	25.37±7.51	25.47±7.00	0.440
	Self-efficacy ^b)^	25.83±8.99	25.23±7.13	0.755

The saliva buffer capacity was measured by comparing the pH average values after adding a colorimetric determination reagent. As a result, the control group showed a pH of 5.74 ± 0.59, and the stress group had a pH of 5.92 ± 0.60, without a significant difference between the two groups (Table 5).

The average saliva secretion level was 1.46 ± 0.67 ml/min in the control group and 1.61 ± 1.69 ml/min in the stress group, without a significant difference between the two groups (Table 5).

Stress awareness survey

Calculating the points resulted in a higher stressor level of the stress group as compared to the control group, but without a significant difference, and there was no difference in stress reaction and self-efficacy (Table 5). The questionnaire was filled out in the classroom by the children themselves and no questions were left unanswered.

Concentration and activity of salivary stress substances

The concentration of salivary chromogranin A (CgA) was measured by enzyme-linked immunoassay (ELISA). The average value of the stress group was more than three times higher with a significant difference (Table 6). When comparing the CgA class by the number of children, class 0 was most common in the control group and class 3 in the stress group (Table 6). There was no significant difference in salivary amylase activity and salivary cortisol concentration between the two groups (Table 6).

**Table 6:** Concentration of stress substance in saliva Mean ± SD

	Control group	Stress group	p-value
CgA (pmol/ml)	2.47±1.75	7.75±10.53	0.005
Amylase (kIU/ml)	89.11±41.87	70.07±53.24	0.088
Cortisol (ng/ml)	0.119±0.0255	0.113±0.0177	0.476

Correlation analysis between caries experience tooth and examination items

The data of all subjects were statistically analyzed whether the results of tested items correlated with the dental caries experience tooth ratio (df and DF tooth ratio). The presence of caries-related microorganisms was calculated as the sum of the presence or absence of the three microorganisms; *Streptococcus Mutans* group, genus *Lactobacilli,* and genus *Candida *(minimum value 0 - maximum value 3).

As shown in Table 7, the df and DF tooth ratio correlated weakly with the class of genus Lactobacilli and the presence of caries-related microorganisms with a coefficient of >0.2. However, *Mutans streptococci*, genus *Candida*, saliva secretion level, and saliva buffer capacity were not correlated.

**Table 7:** Correlation coefficient of 60 children in both groups (*p < 0.05, ** p<0.01)

			df+DF tooth ratio	Mutans streptococci	Lactobacillus	Candida	Caries-related microorganisms	Saliva buffering capacity	Saliva secretion level	Stressor	Stress response	Self-efficacy	CgA class	Amylase class	Cortisol class
Spearman's ρ	df+DF tooth ratio	Coefficient		0.06	0.28(*)	0.18	0.29(*)	-0.03	0.02	0.22	0.20	0.05	0.50(**)	-0.04	-0.02
		Significance probability		0.653	0.031	0.177	0.027	0.832	0.89	0.095	0.143	0.701	0	0.774	0.886
		N		60	60	60	60	60	60	58	58	58	60	58	59
	Mutans streptococci	Coefficient			0.17	0.24	0.48(**)	-0.21	-0.15	0.31(*)	0.17	-0.09	0.13	-0.19	0.17
		Significance probability			0.186	0.071	0	0.113	0.262	0.017	0.199	0.523	0.333	0.158	0.213
		N			60	60	60	60	60	58	58	58	60	58	59
	Lactobacilli	Coefficient				0.28(*)	0.56(**)	-0.04	0.10	0.28(*)	0.14	-0.11	0.16	-0.16	0.05
		Significance probability				0.029	0	0.742	0.453	0.031	0.302	0.426	0.238	0.231	0.716
		N				60	60	60	60	58	58	58	60	58	59
	Candida	Coefficient					0.84(**)	0.18	0.03	0.03	0.15	-0.02	0.22	-0.12	-0.11
		Significance probability					0	0.172	0.807	0.801	0.252	0.873	0.099	0.391	0.429
		N					60	60	60	58	58	58	60	58	59
	Caries related microorganisms	Coefficient						0.02	-0.06	0.20	0.14	-0.03	0.33(**)	-0.13	-0.04
		Significance probability						0.902	0.663	0.14	0.306	0.832	0.01	0.349	0.779
		N						60	60	58	58	58	60	58	59
	Saliva buffering capacity	Coefficient							0.38(**)	-0.28(*)	0.07	0.20	-0.23	-0.07	0.01
		Significance probability							0.003	0.035	0.606	0.13	0.076	0.594	0.936
		N							60	58	58	58	60	58	59
	Saliva secretion level	Coefficient								-0.25	-0.05	0.02	-0.15	-0.03	0.09
		Significance probability								0.062	0.704	0.859	0.262	0.843	0.497
		N								58	58	58	60	58	59
	Stressor	Coefficient									0.57(**)	-0.11	0.31(*)	-0.19	0.18
		Significance probability									0	0.435	0.017	0.17	0.182
		N									58	58	58	56	57
	Stress response	Coefficient										-0.16	0.15	-0.21	0.21
		Significance probability										0.228	0.262	0.119	0.11
		N										58	58	56	57
	Self-efficacy	Coefficient											60.00	249.00	0.13
		Significance probability											0.657	0.065	0.338
		N											58	56	57
	CgA class	Coefficient												0.16	0.08
		Significance probability												0.24	0.54
		N												58	59
	Amylase class	Coefficient													-0.04
		Significance probability													0.785
		N													58
	Cortisol class	Coefficient													
		Significance probability													
		N													

The stressor points correlated weakly with the class of *Mutans streptococci* and genus *Lactobacilli*, and a slight inverse correlation of about -0.3 was observed between stressor points and saliva buffering capacity.

On the other hand, a moderately significant correlation between CgA and the df and DF tooth ratio (ρ = 0.501, p <0.001) was observed. Further, a weak correlation of about 0.3 was observed between CgA and the presence of caries-related microorganisms or stressor items.

The 60 children in the stress and control group were divided into group A with less than six df + DF teeth (40 children) and group B with six or more df + DF teeth (20 children); both groups were compared. Children with caries had an average of 5.3 carious teeth; therefore, the cut-off value was set at six teeth.

CgA in group B (average value 7.81 pmol/ml) was significantly higher than in group A (average value 3.76 pmol/ml) (Table 8).

**Table 8:** Concentration of CgA and df + DF number of teeth in the stress and control group A; df+DF<6 group, n=40, B; df+DF≧6 group, n=20

	A	B	p-value
CgA (pmol/ml)	3.76±4.42	7.81±12.02	0.016

In order to derive an associated factor for the occurrence of df + DF teeth, a binomial logistic regression analysis was performed. Using the forced entry method and setting the dependent variable at ≧6 df + DF teeth, the result showed a large odds ratio (9.04, p <0.05) for the DF index depending on belonging to the stress group or the control group (factor: group) (Table 9). This section may be divided into subheadings. It should provide a concise and precise description of the experimental results, their interpretation, as well as the experimental conclusions that can be drawn.

**Table 9:** Result of binomial logistic regression analysis (variable in the equation) *a Step 1: input variable group, stressor, stress response, self-efficacy, saliva buffering capacity, saliva secretion level, Mutans class, Lactobacilli class, Candida class, CgA_class, amylase_class, cortisol_class Exp (B): Odds ratio ‘Group’ means belonging to the control group or stress group

		B	Standard error	Wald	Degree of freedom	Exp (B)	95% Confidence Interval		Significance probability
							Upper	Lower	
step1(a)	Group	2.49	1.04	5.78	1	12.11	1.58	92.57	0.016
	Stressor	-0.02	0.07	0.06	1	0.98	0.85	1.14	0.809
	Stress response	0.15	0.10	2.83	1	1.17	0.98	1.39	0.093
	Self-efficacy	0.05	0.06	0.77	1	1.05	0.94	1.18	0.381
	Saliva buffering capacity	-1.04	0.80	1.67	1	0.36	0.07	1.71	0.197
	Saliva secretion level	0.11	0.22	0.23	1	1.11	0.72	1.70	0.631
	Mutans streptococci class	-1.66	2.28	0.53	1	0.19	0.01	16.51	0.467
	Lactobacilli class	-0.12	1.01	0.01	1	0.89	0.12	6.38	0.905
	Candida class	0.52	0.56	0.88	1	1.68	0.57	5.00	0.348
	CgA_class	-0.18	0.42	0.19	1	0.83	0.37	1.88	0.660
	Amylase_class	0.41	0.38	1.17	1	1.50	0.72	3.13	0.280
	Cortisol_class	-0.23	0.40	0.32	1	0.80	0.37	1.74	0.572
	constant	-4.38	4.51	0.94	1	0.01			0.333

## Discussion

The purpose of this study was to clarify the relationship between dental caries prevalence and the stress level of children and to compare it with the effect on the oral environment and the microorganisms, known as the dental caries risk factor.

The evaluation of stress is complicated because family-related factors, such as the socio-economic environment, the physical environment, or the mental condition of the mother, also play a role [21]. Therefore we have set multiple evaluation items in this study.

Furthermore, we examined the relationship between stress and dental caries risks, such as cariogenic microbial factors and salivary caries resistance in the oral environment. In the surveyed elementary school, the number of caries experience teeth (number of DF teeth) was much higher than the average of national elementary schools according to the result of the children’s oral health examination over the past 10 years. As a special characteristic of the elementary school, approximately 20% of the children were living in nearby child protection facilities or mother and child dormitories. The return rate of the statement with the recommendation for dental treatment was as low as 71% (2007, data not shown), and in about 30% of the families, children lived in an environment close to neglect and without school attendance.

The commonly used term ‘stress’ mixes up the meaning of external stress stimulus (stressor) and the biological stress response caused thereby. In the subjective part of the survey, the stress stimulus and the stress response were distinguished and evaluated. We also tried to evaluate stress objectively by measuring stress markers in the living body. Several stress-related substances in saliva, which can be biochemically measured, are currently recognized as stress markers [22]. However, the accuracy and sensitivity of marker substances indicating chronic stress are not sufficiently examined and consensus has not been obtained [23]. Measuring the concentration of stress substances such as cortisol in the blood is said to be an objective method [24]. However, measuring these stress markers by blood sampling is painful, resulting in additional mental stress and making accurate evaluation difficult. Therefore, we considered it advantageous to use saliva, which is non-invasive. In this study, we focused on CgA [23], amylase, and cortisol [24], which are considered stress-related substances in many papers, and measured them. CgA is a protein secreted from adrenal medullary chromaffin cells and sympathetic neurons. It is present in the submandibular duct and released into saliva by autonomic nerve stimulation, so it can be used as a mental stress marker [9]. The main source of salivary amylase is the parotid gland and its secretion is due to sympathetic nerve stimulation. Stress is considered the main cause of salivary amylase activity change [25]. Salivary cortisol is secreted when the HPA-system activity is promoted and the brain recognizes stress. This substance is suitable for the detection of short-term stress [26], however, compatibility with long-term stress is still unknown. Other methods evaluate stress by measuring catecholamine in saliva, however, catecholamine is unstable at room temperature [27], and not suitable for measurements at group examinations, therefore it was not used this time. In addition, it has been reported, that the long-term stress of breast cancer patients does not change salivary catecholamine levels [28]. In the present study, oral cavity and oral environment examinations as well as a stress awareness survey and concentration measurement of salivary stress substances (CgA, amylase, and cortisol) were carried out and statistically analyzed.

The results of the df + DF tooth ratio survey showed a significantly higher value for the stress group (Table 4). Comparing the microbial factors between the two groups, it was unexpectedly found that there was neither a difference in the average class value of *Mutans streptococci*, genus *Lactobacilli,* and genus *Candida* (Table 5) nor a strong correlation between microbial factors and the df + DF tooth ratio (Table 7). The single effect of microbial factors on caries development is considered low. Furthermore, saliva secretion level and buffering capacity were not correlated with the df + DF tooth ratio. Therefore, it can be concluded that the stress group has not the generally observed caries risk. On the other hand, the presence or absence of caries-related microorganisms was correlated with the df + DF tooth ratio weakly (Table 7), which seems to be influenced by the existence of genus *Lactobacilli*. Genus *Lactobacilli* reflects the existence of progressive dental caries rather than its cause [29]. The main cause of dental caries is *Mutans streptococci*. Therefore, our data also seems to reflect that the existence of genus *Lactobacilli* results from the existence of active dental caries.

The analysis of the subjective stress awareness indicated no significant difference between the two groups. Because this is a cross-sectional study, it reflects stress at the time of sampling. However, the onset of caries preceded sampling, it is not possible to evaluate long-term stress from the onset of caries.

In Japan, more than 99% of elementary schools conduct questionnaire surveys on bullying, but only 54.2% find increasing numbers of bullying and suicides. Although anonymous questionnaires regarding bullying are generally preferable, about 70% of schools request the names in order to determine who is being bullied. Therefore, children who are bullied are unlikely to answer honestly because they fear that bullying will worsen by admitting that they are being bullied. In this regard, questionnaires indicating the child’s name have many problems and are considered unreliable [30]. Therefore, the frequency of visits to the infirmary for long-term psychogenic counseling as well as the verification of nursing teachers who observe children over a long period and cooperate with other teachers and parents, are considered important components. In this study, 30 children were selected using these two components as stress indicators.

Among the stress substances, salivary amylase activity and cortisol concentration did not differ between the two groups, but the CgA concentration was significantly higher in the stress group (Table 6). Comparing the CgA class depending on the number of children, it seemed that the stress state is reflected because the control group had the most children in class 0 while the stress group had the most in class 3 (Table 3). Therefore, in the groups with df + DF teeth <6 and df + DF teeth ≥ 6, the latter group had a significantly higher CgA (Table 9).

However, in the binomial logistic regression analysis with the df + DF tooth ratio as the dependent variable, the involvement of CgA was not significant. Belonging to the stress group was the only significant factor showing a large odds ratio (Table 9).

Therefore, it is suggested that being in a stress state can be associated with an increased caries risk.

Insufficient oral care may be due to the deterioration of the living environment of the stress group, and it is said that the educational and socio-economical background of the parents is particularly related to the oral hygiene management status of the children [6]. However, in Japan, if dental treatment becomes necessary for children in a child protection facility, visits to a dental clinic are overseen by the supervisor. Further, due to tooth-brushing guidance on an individual level at the elementary school, oral hygiene disparity is considered low. Besides, many cities in Japan, including the surveyed elementary school offer free dental treatment and precautional measures for children, eliminating the economic burden for parents. Nonetheless, in this study, differences between *Mutans streptococci* and genus *Candida* could not be detected (Table 5), so the development of dental caries is unlikely to be directly caused by a lack of oral hygiene in the stress group.

In past research, stress was found to be a possible dental caries-causing factor. We examined whether the reasons also apply to the results of this study.

Stress is believed to increase catecholamines and corticosteroids in blood serum and saliva to affect the immune system and impair the host’s resistance to cariogenic bacteria. However, dental caries is widely known as a pathological change that does not recover by immunity. Also, as there were no systemic or infectious diseases in children of the stress group, it seems that immunity was not extremely reduced. It is known, that acute stress decreases salivary secretion and, as a consequence, the clearance of cariogenic bacteria. However, this phenomenon is unlikely to occur in the case of chronic stress. In fact, our results showed neither a difference in saliva secretion levels nor a difference in the detected amount of *Mutans streptococci* between the two groups. To disperse or release stress an emotional and unhealthy eating habit of increasing the intake of snacks and meals containing sugar is thought to be a cause of caries. However, since many children in the stress group lead a properly managed daily life at the facilities, it seems that there are no unhealthy eating habits. Data are not shown here, but about half of the subjects in this study were asked about the frequency and type of sucrose-containing snacks they eat between meals. The two groups were compared and the stress group tended to consume rather less, which shows that diet management is sufficient. In a stress-prone living environment, oral self-care habits tend to fail, leading to impaired oral hygiene. As a result, the oral cavity becomes a favorable environment for cariogenic bacteria. However, in this study, it seems that there is no problem with oral care because it is properly managed, and the influence of microbial factors on dental caries development is considered low. In fact, our results of caries-related microbial tests did not show any difference between the stress and control groups.

For these reasons, it is possible that the theories previously reported do not apply to young children regarding how stress is responsible for caries susceptibility. It is known that the degree of maturity of living organisms declines due to psychological stress, for example, affection deprivation dwarfism. In the same way, it might be possible that a lower maturity degree of teeth affects their caries resistance. Future research is needed to find out what the actual factors are.

In the future, it is necessary to develop a simple and sensitive method for judging a child’s stress to assess not only the health of the oral cavity but also the health of the whole body and mind.

## Conclusions

Child abuse and bullying are serious problems in the world and it is important to discover them at the earliest possible. The results of our study revealed that even after adjusting for the effects of the biological status (amount of cariogenic bacteria, salivary flow, saliva buffering capacity), being in the stress group was significantly associated with more caries. The most notable finding among those statuses was that we did not detect any differences in rates and counts of cariogenic bacteria or salivary buffering activity within the stress and control groups. Therefore, long-term stress, causing changes in the children's bodies, might hint at important factors leading to developing dental caries. Future research is necessary to elaborate on the influencing mechanisms.
